# NMN Rescues Endothelial Function and Neurovascular Coupling, Improving Cognitive Function in Aged Mice

**DOI:** 10.1093/geroni/igaa057.399

**Published:** 2020-12-16

**Authors:** Stefano Tarantini, Andriy Yabluchanskiy, Praveen Ballabh, Eszter Farkas, Joseph Baur, David Sinclair, Anna Csiszar, Zoltan Ungvari

**Affiliations:** 1 University of Oklahoma Health Sciences Center, Oklahoma City, Oklahoma, United States; 2 Biochemistry and Molecular Biology, Oklahoma City, United States; 3 Albert Einstein College of Medicine, Bronx, NY, United States; 4 Faculty of Medicine, Szeged, Hungary; 5 Perelman School of Medicine at the University of Pennsylvania, Philadelphia, Pennsylvania, United States; 6 Harvard Medical School, Boston, United States; 7 University of Oklahoma Health Sciences Center, Oklahoma City, United States

## Abstract

Adjustment of cerebral blood flow (CBF) to neuronal activity via neurovascular coupling (NVC) has an essential role in maintenance of healthy cognitive function. In aging increased oxidative stress and cerebromicrovascular endothelial dysfunction impair NVC, contributing to cognitive decline. There is increasing evidence showing that a decrease in NAD+ availability with age plays a critical role in a range of age-related cellular impairments but its role in impaired NVC responses remains unexplored. The present study was designed to test the hypothesis that restoring NAD+ concentration may exert beneficial effects on NVC responses in aging. To test this hypothesis 24-month-old C57BL/6 mice were treated with nicotinamide mononucleotide (NMN), a key NAD+ intermediate, for 2 weeks. NVC was assessed by measuring CBF responses (laser Doppler flowmetry) evoked by contralateral whisker stimulation. We found that NVC responses were significantly impaired in aged mice. NMN supplementation rescued NVC responses by increasing endothelial NO-mediated vasodilation, which was associated with significantly improved spatial working memory and gait coordination. These findings are paralleled by the sirtuin-dependent protective effects of NMN on mitochondrial production of reactive oxygen species and mitochondrial bioenergetics in cultured cerebromicrovascular endothelial cells derived from aged animals. Thus, a decrease in NAD+ availability contributes to age-related cerebromicrovascular dysfunction, exacerbating cognitive decline. The cerebromicrovascular protective effects of NMN highlight the preventive and therapeutic potential of NAD+ intermediates as effective interventions in patients at risk for vascular cognitive impairment (VCI).

